# An Online Tool to Assess Sentence Comprehension in Teenagers at Risk for School Exclusion: Evidence From L2 Italian Students

**DOI:** 10.3389/fpsyg.2019.02417

**Published:** 2019-11-05

**Authors:** Mirta Vernice, Michael Matta, Marta Tironi, Martina Caccia, Elisabetta Lombardi, Maria Teresa Guasti, Daniela Sarti, Margherita Lang

**Affiliations:** ^1^Department of Psychology, University of Milano-Bicocca, Milan, Italy; ^2^Department of Psychological, Health and Learning Science, University of Houston, Houston, TX, United States; ^3^Scientific Institute IRCCS Eugenio Medea, Bosisio Parini, Italy; ^4^Department of Psychology, Catholic University of the Sacred Heart, Milan, Italy; ^5^Foundation IRCCS Carlo Besta Neurological Institute, Milan, Italy; ^6^A.R.P. Associazione per la Ricerca in Psicologia Clinica, Milan, Italy

**Keywords:** sentence comprehension, L2 speakers, adolescents, school exclusion, online tool

## Abstract

This study presents a web-based sentence comprehension test aimed at identifying high school students who are at risk for a language delay. By assessing linguistic skills on a sample of high school students with Italian as an L2 and their monolingual peers, attending a vocational school, we were able to identify a subgroup of L2 students with consistent difficulties in sentence comprehension, though their reading skills were within the average range. The same subgroup revealed to experience a lack of support within the school context, suggesting that poor L2 skills might be a critical variable to consider in order to identify students at risk for school exclusion. Regarding the test, accuracy to the on-line sentence comprehension task was significantly predicted by reading abilities and vocabulary skills, thus indicating that this test might represent a rapid but efficient way to assess linguistic abilities at school. We recommend that establishing a valid and practical procedure for the evaluation of linguistic skills in bilingual students who struggle with their L2 is the first step toward promoting social inclusion in the multilingual classroom, in order to increase their ability to actively participate in school and social activities.

## Introduction

The Italian educational system is currently undergoing a significant change toward promoting inclusion of students coming from the most diverse backgrounds. To this aim, in the last few years, a tremendous transformation took place in terms of digital technology enhancement in the classroom. According to the national educational policy known as “The Italian National Plan for Digital Education” [Piano Nazionale Scuola Digitale] (Italian Ministry of Education [MIUR], 2015b), digital technology deployment in the classroom is aimed at fostering student engagement by creating a learner-centered environment that promotes inclusion of all students, in particular those with special needs (Italian Ministry of Education [MIUR], 2015a, b). However, in the very same years, the rapid growth in the number of students with learning difficulties of the most heterogeneous nature (e.g., specific learning disorders; atypical language acquisition; language or cultural deprivation) had a critical impact on the schooling system making it difficult to foresee educational programs able to integrate and include the greatest number of pupils and in particular those at risk of school exclusion (Contini, 2013).

Moving from the assumption that, in the digital classroom, teachers must be equipped with online instruments able to identify those students who might show a learning or linguistic problem that might prevent their full integration within the classroom, we present a web-based tool that allows discriminating those students that belong to a population at potential risk for a language problem, and thus, for school exclusion: teenagers speaking Italian as an L2 (henceforth L2 students).

### L2 Students: A Population at Risk for School Exclusion

In the last years, consistent growth in the number of immigrants has caused profound modifications in the Italian educational system. According to data from the Italian Ministry of Education [MIUR] (2014, 2017), the number of L2 students increased from 60,000 in 1997 to over 800,000 in 2016 (excluding universities). Crucially, in 10 years, the rate of L2 students attending a high school increased in percentage significantly more in comparison with other school levels. In fact, from 2007 to 2017, there were 82% more L2 students in high schools, as compared to lower secondary schools (45% more), primary schools and kindergartens (56% and 76%, respectively) (Italian Ministry of Education [MIUR], 2017). Considering only high schools, national reports indicate a total of 23% of immigrant students (including both first- and second-generation immigrants), with consistent differences across regions and type of high school (Italian Ministry of Education [MIUR], 2017). For instance, out of the total number of L2 students attending a high school, 92% of them, in 2016/2017, opted for a vocational-technical school. Additionally, Lombardy, the region where the current research took place, appears to be characterized by the highest presence of L2 students (25%) in high school classrooms with respect to the national average.

A recent report by the National Institute of Statistics (ISTAT, 2016) further indicates that L2 Italian students appear to be significantly more vulnerable than monolingual students in terms of academic failures. They tend to achieve lower academic outcomes, with higher proportions of dropouts and lower-levels of school attainment (ISTAT, 2016). Some studies based on Italian data (Murineddu et al., 2006) went further by indicating that low academic achievements in this population are associated with learning problems. Research has shown that the presence of L2 students in the classroom might exert an adverse effect on the well-being and inclusion in the classroom. For instance, Brunello and Rocco (2013) analyzed to what extent the presence of immigrant students exerts an impact on the school performance of L1 students at 15 years of age, finding evidence of a significant negative effect, increasing with the level of segregation of immigrants, as evidenced by larger dropout rates.

A number of previous studies investigated the difficulties of the educational pathway of immigrant students in Italy, by addressing the multiple factors that might concur in determining such condition. For instance, Mussino and Strozza (2012) presented a national survey (ITAGEN2) involving more than 20,000 lower-secondary school students, half of whom were L2 speakers. Starting from the observation that L2 students showed higher drop-out rates and fewer academic achievements, the survey allowed to identify a number of possible factors causing a lack of integration of immigrant students in the Italian school system, among which the authors included educational delay and socioeconomic deprivation. However, lack of school integration was significant even when immigrant students showed comparable socioeconomic and housing conditions in comparison with their Italian peers. The authors concluded that it was mainly socialization with peers that appeared to decrease the risk of isolation, facilitating a faster inclusion in the school context.

As for the role of academic and learning skills, a recent Italian study observed that students who are struggling in reading and writing, regardless of whether they were L1 or L2 speakers, developed low motivation with respect to their abilities, helpless behavior and anxiety in being involved in school activities (Andolfi et al., 2015). Again, this study confirmed that only support within the peer group reduced the risk of school failure and, most importantly, the feeling of exclusion.

The above-mentioned findings suggest that when studying the possible causes of school exclusion, it is crucial to consider not only aspects related to the socioeconomic level and learning profile, but also factors such as students well-being, including relationships with classmates, engagement, and support from peers (Deci and Ryan, 2008).

Interestingly, to our knowledge, relatively little research has directly addressed the role of L2 proficiency in promoting school inclusion of L2 students. Indeed, while it is uncontroversial that being proficient in the language of instruction represents, in general, a protective factor for L2 students (cf., in primary school, Whiteside et al., 2017), it is not yet consolidated that poor L2 skills might be directly related to risk of school exclusion in high school students. In the current study, we hypothesize that L2 proficiency of immigrant teens, tested at school by means of an *ad hoc* created test of sentence comprehension, could be regarded as a critical variable in order to identify those students that might experience difficulties in their educational pathway. That is, we propose that L2 students’ proficiency in the language of instruction might offer valuable information to signal those students who could be at risk for school exclusion.

Before we move on to describe the test used to investigate language proficiency in L2 teens, we need to discuss the profile of bilingualism at stake in the current study, taking into consideration some factors, such as the cultural and socioeconomic background (i.e., socioeconomic status, SES) as well as the age of exposure to the L2 of bilingual students. In fact, in the current study, we are concerned with L2 students who come from a context of relative socioeconomic disadvantage and that were exposed to Italian since birth (except for two, who came to Italy when they were infants).

With regards to the SES, in Italy, a growing number of L2 students comes from immigrant families that live below the poverty level and have, on average, lower-levels of education compared to non-immigrant families (ISTAT, 2016). Research suggests that in monolingual development, students who come from a deprived context tend to be exposed to a less varied and less rich linguistic input than that of children from high SES families (Myers and Botting, 2008). The same applies to immigrant children living in a context of relative socioeconomic disadvantage: L2 children often begin schooling without being able to speak the majority language and then start acquiring it during the preschool years (Spencer et al., 2012). Usually, a child becomes dominant in his/her L2 only when formal education begins, while dominance in other languages decreases (Montrul, 2008, 2015; Benmamoun et al., 2013; Puig-Mayenco et al., 2018). However, research indicates that many of them acquire a limited L2 proficiency to catch up to their monolingual peers. Crucially, in such a case, language problems cannot be attributed to bilingual development, rather to the deprivation in terms of language input (Spencer et al., 2017). Note indeed that studies conducted in countries where bilingualism is actively promoted (such as Canada), show that bilingual children are not at risk for a language problem, rather, that (early) exposure to two languages is related to several cognitive benefits (Bialystok, 1999; Bialystok and Martin, 2004; Bialystok and Feng, 2009; Luk et al., 2011; Paradis et al., 2011; Poarch and Bialystok, 2015; also with immigrant children see Robinson and Sorace, 2019).

A second factor in determining bilingual language outcome is the age at which children are first exposed to each language. According to the literature, children who are exposed to two languages from early infancy (the so-called “early bilinguals”) generally achieve greater proficiency than speakers exposed to a language after 3 years of age (Flege et al., 1999; Kovelman et al., 2008). However, as Unsworth (2014, 2016) notes, what is crucial is not only the length of exposure to the L2 input but also the quality and the real amount of language use. For instance, L2 children are often exposed to input from both native and non-native L2 speakers. However, predominant exposure to non-native speakers is often associated with poorer levels of language proficiency. Therefore, it is important to consider the fact that even pupils that are exposed to Italian since birth might show a language delay in L2.

In the current study, we involved a sample of L2 participants as homogeneous as possible in terms of age of first exposure to the L2. We are aware of the fact that, by controlling this variable, the number of L2 participants was limited. As a consequence, the current study should be considered as an initial contribution aiming at characterizing the linguistic competence of early bilingual teens, coming from a deprived socioeconomic context.

### Sentence Comprehension: A Pivotal Ability Underlying Higher-Level Comprehension Processes

The ability to comprehend a syntactically complex sentence is a language skill purporting a variety of activities that characterize high school programs, such as reading complex passages, summarizing them, creating well-structured texts that effectively communicate the content. In adolescence, once the decoding process in reading is automatized, lower-level comprehension skills (i.e., receptive vocabulary and grammar) appear to support the ability to make inferences about the internal structure of complex texts (Gough and Tunmer, 1986) as well as about its lexical content (Kieffer and Box, 2013). That is, lower-level comprehension processes (related to vocabulary and grammar) facilitate the extraction of semantic and syntactic information that supports reading comprehension of connected text (van Gelderen et al., 2007). Thus, language comprehension appears as a critical prerequisite of text reading comprehension not only at sentence-level but also at text-level, promoting lexical and grammatical inference, and thus supporting academic achievement in later grades of school (Kamhi and Catts, 2012).

In L2 students, research suggests that even though typically developing L2 students show appropriate word reading skills since the very first years of primary school, their reading comprehension scores fall below average with respect to their peers (Menken, 2008). Along the course of development this difference in text reading comprehension increases in the upper-grade levels (Galindo and Reardon, 2006). For this reason, being able to identify those students who struggle with language comprehension is critical to the task of creating high-quality academic education and effective inclusion strategies.

Up-to-date research has focused mostly on children and there has been little investigation into reading comprehension skills of L2 adolescents. In general, assessment of language skills in adolescence is difficult due to the lack of standardized tests for this age group in Italian. Additionally, according to Italian data regarding language and communication difficulties, adolescents are rarely assessed for previously undetected language problems (Brizzolara et al., 2011). This study, therefore, represents one first attempt to identify language difficulties in Italian L2 teens, by using an online sentence comprehension text. To this aim, we created a task that was based on 20 multiple-choice trials. Each trial involved a target sentence of varying syntactic complexity that had to be read silently. Then four additional sentences followed. One was a paraphrase of the target sentence, equivalent in meaning to the target sentence; the second sentence contradicted the meaning of the target sentence; the third one involved a meaning compatible with the target sentence, but not equivalent; the fourth one was a distractor involving a different content. Correct responses were based on the number of equivalent sentences detected. The sentence comprehension test was presented using Google forms. The choice of a web-based tool, instead of a canonical paper and pencil test, moved from the consideration that L2 adolescents tend to show low levels of participation and involvement to research (Myers and Botting, 2008; Clegg et al., 2009), suggesting that this population may be hard to reach and to be involved in projects. Digital technology might come in hand, being a particularly useful tool for motivating learning (Zhang, 2008). In particular, many observations about motivating L2 students in classroom situations support the idea that digital technology provides “motivational affordances” (ibidem). Students show a feeling of intrinsic motivation in performing an online task when they experience competence, autonomy and social connection (Ryan and Deci, 2000). Therefore, in such a case, the opportunity to use a digital device to run the assessment would result in a more committed participation in the study.

To sum up, the current study aims: (1) to compare the performance of L1 and L2 students on a series of standardized tests as well as on a web-based assessment of sentence comprehension; (2) to identify the profile of L2 students whose performance on the sentence comprehension assessment proved to be insufficient to allow the student to actively participate in school activities. In general, this study aimed at promoting the use of a web-based tool in order to facilitate the identification of subjects at risk for a language problem. In fact, by gaining further information about the type of L2 linguistic development of the student and the eventual occurrence of language difficulties, teachers might be able to effectively supply to the needs of students.

## Materials and Methods

### Participants

A total of 44 teenagers who attended a vocational high school in the region of Lombardy (Italy) participated in this study. Participants ranged in age from 14;9 (years; months) to 17;4 (Mean age in months = 189.66, SD = 7.09). Participants were divided into two groups: L1 Italian students (henceforth L1 group; n = 22, 3 boys; Age range: 14;9 – 17;1; Mean age in months = 189.59, SD = 6.90) and L2 Italian (L2; *n* = 22, 3 boys; Age range: 14;10 – 17;40; Mean age in months = 189.73, SD = 7.43), matched for gender and chronological age (*t* = −0.063, *p* = 0.950).

First languages of L2 students were Chinese (3), Arabic (7), Spanish (1), Albanian (3), Roumanian (4), Punjabi (4). Twenty of them were born in Italy, while the remaining two came to Italy during early infancy (i.e., before than 2 years of age; Mean total exposure to Italian in months = 182.63, SD = 23.47). L2 students could thus be considered early bilinguals (Kovelman et al., 2008). All participants received their formal education only in Italy.

To be eligible to participate, students had to meet a number of criteria. First, they reported no history of learning, cognitive, neurological, or sensory disorders. Second, their cognitive level fell within the normal range (above the 25th percentile). Third, they did not receive any special educational support (according to school reports) because of language problems.

Written informed consent was obtained from the parents of all participating students. The protocol was approved by the Ethical Committees of the University of Milano-Bicocca and of the Catholic University of the Sacred Heart.

### Materials

To address our research questions, participants took part in an online assessment of sentence comprehension. Additionally, they were administered a battery of standardized tests of cognitive level, reading fluency and comprehension, vocabulary, and SES.

### Standardized Tests

Participants were administered a series of tasks from standardized tests, assessing, respectively: (a) non-verbal cognitive level; (b) receptive vocabulary (The Peabody Picture Vocabulary Test Form L; PPVT); (c) reading fluency and passage reading comprehension; (d) SES; (e) level of bilingual exposure (only for the bilingual group); (f) psychological well-being (CIT). Table 1 offers a summary of the different factors that concur in determining a condition of vulnerability at school, namely, L2 proficiency and dominance, learning profile, SES, psychological well-being. On each row, we report constructs investigated and tests used. Tests and questionnaires are described below.

**TABLE 1:** Summary of the different factors that were considered in order to evaluate the risk of school exclusion in our sample, and related tests that were used to measure each factor.

**Factors**	**Skills and constructs evaluated**	**Tests**
L2 Proficiency and use	L2 Receptive vocabulary, dominance and use of L2, L2 sentence comprehension	PPVT, LSBQ, Sentence comprehension assessment
Learning profile	Reading fluency and comprehension	MT 3 Advanced (passage reading comprehension), DDE-2 (word and non-word reading)
Socioeconomic status	Family wellness	Family Affluence Scale (FAS)
Psychological well-being	General well-being including engagement, quality of relationships and support from peers	Comprehensive Inventory of Thriving (CIT)

To investigate the non-verbal cognitive level, we used the Raven’s Progressive Matrices Test (SPM version; Raven and Court, 1998). SPM measures fluid intelligence (Horn and Cattell, 1966) which correlates strongly with IQ scores (Kvist and Gustafsson, 2008) and involves low degrees of cultural loading and linguistic demand (Flanagan et al., 2013). We used this measure to the extent of identifying participants whose cognitive non-verbal level was within the normal range (above the 25th percentile).

Vocabulary skills were assessed by means of The Peabody Picture Vocabulary Test Form L (PPVT, Dunn and Dunn, 1981). The primary aim of this test is to measure the receptive vocabulary for standard Italian. Note that as norms for the Italian population are not yet available, we conducted analysis only on raw scores.

In order to evaluate bilingual exposure and dominance, we opted for a sensitive instrument that provides a comprehensive description of bilingualism that applies to a broad range of contexts: the Language and Social Background Questionnaire (LSBQ; Anderson et al., 2018). A copy of the Italian version of the questionnaire is available for view at the following link: https://drive.google.com/file/d/1VVubXIjaLq9-Ep_KN-CsmsekzPWf04kt/view?usp=sharing. The questionnaire includes a detailed description of bilingual usage patterns across different situations. The questionnaire provides a composite factor score that represents the overall level of bilingualism. Critically, this composite score can be used as both a continuous variable or as a cut-off to discriminate groups categorically. According to the authors, students with a composite score of less than −3.13 could be classified as monolinguals (in that one language was significantly more dominant than the other), whereas participants with a composite score above 1.23 are considered bilinguals. It is important to note that information about language dominance in such sample needs to be considered with caution. Indeed it could be possible that an L2 student could be classified as monolingual because s/he mostly uses Italian in everyday life, but it is also possible to be regarded as monolingual being more dominant in the L1. Note that the information about language dominance could be used both categorically and continuously. In the current paper we considered the score as a cut-off in order to discriminate between those L2 students that were dominant in their second language versus those that were dominant in their L1.

Regarding the learning profile, word and non-word reading fluency, and passage reading comprehension scores were obtained through the administration of the following Italian standardized tests: (1) “Test of word and non-word reading” drawn from “Batteria per la valutazione della dislessia evolutiva DDE-2” [Battery for the Assessment of Dyslexia and Developmental Dysorthography 2] (Sartori et al., 2007). Norms for high school students were based on the study of Arina et al., 2013; (2) “Passage reading comprehension test” drawn from “Prove MT Avanzate-3-clinica” ([MT 3 Advanced], Cornoldi et al., 2017), which provides accuracy scores for passage reading comprehension.

As for the third factor, SES, we administered the Family Affluence Scale (FAS; Currie et al., 2008) to identify students’ SES. The questionnaire collects information about family wellness.

Psychological well-being was assessed through the Comprehensive Inventory of Thriving (CIT; Su et al., 2014), a validated self-report questionnaire. It is composed by 54 items assessing 18 facets of positive functioning that represent seven dimensions of psychological well-being. The responses to each item vary on a Likert-scale from 1 (“Strongly Disagree”) to 5 (“Strongly Agree”). In addition to total scores, we also considered eighteen sub-scales: Support, Community, Trust, Respect, Loneliness, Belongingness, Flow, Skill, Learning, Lack of Control, Accomplishment, Self-efficacy, Self-worth, Meaning, Optimism, Life satisfaction, Positive emotions, and Negative emotions. Note that, even though the CIT is not directly meant to offer an indication of school exclusion, it provides a reliable and valid multi-component measure of well-being in a social context. In particular, we put the attention to the Support scale, which investigates the perception of how much others, as well as the external context, act as a support for the subject.

### Sentence Comprehension Assessment

In order to evaluate the proficiency in L2 sentence comprehension, we created a simple test aimed at evaluating the ability of teen students to comprehend complex sentences. The sentence comprehension assessment involved 20 multiple-choice items made up of a target sentence and four possible options. In order to create a set of sentences for the sentence comprehension assessment, we proceeded as follows. First, we selected several items drawn from the well known psychodiagnostic tests MMPI-RF (Ben-Porath and Tellegen, 2008; 46 items), PAI (Morey and Boggs, 1991; 21 items) e DAPP-BQ (Livesley and Jackson, 2009; 16 items). Sentences were chosen with respect to their syntactic complexity: they might involve a subject or an object relative clause (Traxler et al., 2002; Staub, 2010; Yang, 2013), a double negation within the same NP (i.e., a negative reversal structure, Chierchia, 2013), a hypothetical conditional or multiple subordinate clauses. We provide below an example of a sentence drawn from the MMPI-RF that was selected, followed by the English equivalent:

(1) Non sarei preoccupato se qualcuno dei miei famigliari si trovasse nei guai con la legge.
‘It wouldn’t make me nervous if any members of my family got into trouble with the law.’

In general, we selected structures that were regarded in the literature to cause slower processing (Grodner and Gibson, 2005) or difficulty in anticipating the structure (Levy, 2008).

We then asked 44 native Italian adults (age range: 19–43, 7 M), to rate the grammatical acceptability of the 83 sentences so identified on a 1 to 5 Likert scale. By doing so, we selected 20 sentences that were rated as less acceptable: 11 of them were drawn from the MMPI-RF, eight from the DAPP-BQ, one from the PAI.

To create the target sentences of the sentence comprehension test, for each sentence, we changed the lexical content, maintaining unaltered the syntactic structure. Thus, (1) was transformed into (2):

(2) Elena non sarebbe tranquilla se qualcuno dei suoi amici si trovasse in difficoltà con un esame.
‘Elena could not relax if any of her friends were in trouble with an exam.’

We further developed four types of response options starting from the original target sentence. The first option was a paraphrase of the target sentence with equivalent meaning (henceforth, “equivalent”, see 2a below). The second type of sentence involved a contradictory meaning with respect to the target sentence (“contradictory”; 2b); the third one presented a meaning compatible with the target sentence, but not equivalent (“compatible”; 2c); the fourth option involved an unrelated content (“distractor”; 2d). Participants were asked to identify the sentence with the same meaning of the target sentence. Correct responses therefore refer to the number of equivalent sentences. We are aware of the fact that several procedures might be developed for scoring the sentence comprehension test. In the current study, we opted for the most straightforward procedure, namely, to compute proportions of correct responses, i.e., the raw number of equivalent sentences identified. The full list of target sentences and the four response options are reported in Supplementary Appendix A.

(2a) Se un amico di Elena facesse fatica con un esame, lei sarebbe in pensiero per lui.
‘If a friend of Elena were struggling with an exam, she would be worried about him.’
(2b) Se tra gli amici di Elena ce ne fosse qualcuno in difficoltà con un esame, a lei non importerebbe molto.
‘If any of Elena’s friends were in trouble with an exam, she would not care much.’

(2c) A Elena importa sapere che i suoi amici stanno bene.
‘Elena cares about her friends being fine.’
(2d) Se uno si trovasse in difficoltà con un esame non potrebbe essere tranquillo.
‘If one were struggling with an exam, he could not relax.’

The sentence comprehension test was presented using Google forms. The test began with a statement outlining the overall purpose of the research. After a short demographic section, participants were asked their reading habits through several short multiple-choice questions. After a couple of practice trials, the test began. Participants had no time limit, and they could go back to the previous sentence whenever they wanted. The test ended by providing the subject with the number of correct responses obtained.

### Procedure

The purpose of the study was explained to all potential participants in an assembly. Participants were provided with an information sheet and consent form to give to their parents. The signed consent form was returned to school, where it was collected. Participants also completed a consent form.

The assessment took place at different times of the school year, in individual and collective administering sessions. Some tests were presented individually in a quiet room (i.e., reading tests); others were proposed through a collective administration (i.e., syntactic assessment and text comprehension, FAS and LSBQ). All tests were administered by qualified psychologists. At the beginning of each session, participants were given the opportunity to ask any questions and to withdraw from the study if they wished. Students were tested in three collective sessions lasting approximately 30–40 min each and in one individual session. As for collective assessments, tests were proposed in the following order: SPM Raven and MT-3 during the first session; Peabody and Sentence Comprehension Test during the second; CIT, FAS, and LSBQ (only for bilingual students) in the third session. Reading fluency, tested through the DDE-2 word/non-word reading task, was individually assessed. Administration of the Sentence Comprehension test lasted about 15 min.

## Results

### Comparison of L1 vs. L2 on Standardized Tests

We provide a short summary of the statistical results comparing L1 vs. L2 students on Table 2. Descriptive statistics (Means, M and Standard Deviations, SD) for all the standardized tests included in the study are reported on Table 2. We compared the performance of L1 vs. L2 students on standardized tests by means of a series of independent samples comparisons (*t*-tests). For simplicity sake, as regards to the CIT test, we report the only Scale that resulted significant, namely Support.

**TABLE 2:** Vocabulary, reading and socioeconomic assessment (mean and standard deviations, independent sample *t*-tests, *p*-values and Cohen’s *d*) for L1 and L2 students on the standardized tests.

**Assessment**		**Mean**	**SD**	***t* (42)**	***p***	**Cohen’s *d***
Peabody (raw scores)	L1	149.59	20.34	1.476	0.147	0.445
	L2	138.68	28.08			
Passage comprehension (*z* scores)	L1	0.50	0.78	2.439	0.019^∗^	0.735
	L2	0.00	0.56			
Word reading accuracy (*z* scores)	L1	0.02	0.86	2.041	0.048^∗^	0.615
	L2	–0.52	0.88			
Word reading speed (*z* scores)	L1	0.18	0.76	2.233	0.031^∗^	0.673
	L2	–0.41	0.98			
Non-word reading accuracy (*z* scores)	L1	0.04	1.01	0.704	0.486	0.212
	L2	–0.17	0.99			
Non-word reading speed (*z* scores)	L1	0.08	0.84	1.499	0.141	0.452
	L2	–0.38	1.16			
FAS (log-transformed)^1^	L1	7.04	1.17	4.116	0.049^∗^	
	L2	5.71	1.49			
CIT (Support)^1^	L1	4.00	0.47	6.567	0.014^∗^	
	L2	3.51	1.04			

*Note that, as regards to the FAS and the CIT (Support scale), the assumption of homogeneity of variance was not supported, we conducted a Levene’s test. ^1^Test of Equality of Variances (Levene’s) used because of the violation of the equal variance assumption. ^∗^Significant at the 0.05 level (two-tailed).*

As shown in Table 2, there were some differences between the two groups regarding word reading fluency, passage comprehension, SES and psychological well-being. As for reading scores, it is important to note that, though appearing within the range of normality (recall that none of the participants were reported to school either for a learning disorder, or for a special educational need), word reading was significantly slower and less accurate in L2 students than in the L1 group. It is important to note that we did not find the same pattern in non-word reading. In such a case, L2 did not differ with respect to L1 readers.

Also passage reading comprehension scores indicated a poorer performance of the L2 students. However, again, L1 outperformed their L2 peers, though showing scores within the range of normality. Differential occurrences in the category of performance to the passage reading comprehension test (i.e., Need for immediate intervention; Attention is needed; Sufficient performance; Complete performance; see Table 3) attested significant across groups differences [χ^2^(3) = 13.23, *p* < 0.004]. Interestingly, 77% of L2 students showed an average performance, while 59% of L1 students involved an optimal performance. As regards to vocabulary skills, we did not find any difference in the raw scores of Peabody. Descriptive data indicate that L1 students were slightly better as compared to their L2 peers, however, such difference was not significant.

**TABLE 3:** Contingency table for the performance categories to the passage reading comprehension test by group.

	**NI**	**AN**	**SP**	**CP**	**Total**
L1	0(0%)	2(9%)	7(32%)	13(59%)	22(100%)
L2	1(5%)	2(9%)	17(77%)	2(9%)	22(100%)
Total	1(2,5%)	4(9%)	24(54%)	15(34%)	44(100%)

*Performance categories refer to NI, need for immediate intervention; AN, attention is needed; SP, sufficient performance; CP, complete performance.*

Scores at the FAS test differed across groups: although students were all drawn from the same school, living in an area of relative socioeconomic disadvantage, we did find a difference in terms of SES between the two groups. Finally, a significant difference was also found with respect to the Support scale in the CIT: L2 were less likely to feel supported by the school context as compared to their L1 peers. Overall, these findings provide evidence for the fact that the L2 group might be considered a sample at risk for school exclusion. In fact, both socioeconomic as well as personal well-being factors seem to concur to draw an unsettling scenario for these pupils.

With respect to the LSBQ, by examining the composite score, only 10 of the L2 participants could be considered “bilingual” (composite score above 1.23). The remaining 12 students were classified as “monolingual” (showing a composite score of less than −3.13; *N* = 6) or “undifferentiated” (composite score falling between −3.13 and 1.23; *N* = 6). Note that the LSBQ provides information about language dominance in terms of patterns of preferred use of and exposure to L1 and L2 in everyday life. Therefore one should not infer anything with regard to proficiency. In general, the inspection of additional factors such as “L1 Home Use and Proficiency” and “L2 Social Use” revealed that most of the participants showed conditions of an unbalanced bilingualism that allowed us to categorize L2 “monolinguals” and “undifferentiated” as dominant in L2 (henceforth “L2-dominant”). L2 students classified as “bilinguals”, although proved to use both languages in their everyday life to a greater extent that L2 “monolingual” peers, revealed a prevalent tendency to use their L1 in most social and personal activities. For this reason we defined such group as “L2-non-dominant”.

### Comparison of L1 vs. L2 on the Sentence Comprehension Test

Descriptive statistics (Means and Standard Deviations, SD) of proportions of correct responses to the sentence comprehension task by group are reported in Table 4 (first section). There were slightly more correct responses (7%) in the L1 group as compared to the L2 one.

**TABLE 4:** Mean proportions and standard deviations (SD) of correct responses to the sentence comprehension test by group (L1 vs. L2) and by group as defined by the LSBQ (L2-dominant vs. L2-non-dominant).

	***N***	**Mean**	**SD**
L1	22	0.90	0.30
L2	22	0.83	0.37
L2-dominant	12	0.87	0.33
L2-non-dominant	10	0.78	0.41

Data analyses were conducted using R (R Core Team, 2015). Results obtained in the sentence comprehension test were analyzed with mixed-effects models, using the “lmerTest” package (Kuznetsova et al., 2017). The dependent variable was fitted to a series of mixed effects models. As accuracy to the sentence comprehension test was a binary variable we used the “binomial family”. In each model, we first tested whether the fixed effects of group (L1 vs. L2) and group based on the LSBQ questionnaire categories (L1 vs. L2-non-dominant vs. L2-dominant) contributed to the model’s fit. We examined whether fixed and random effects added significant information to the model by means of a series of likelihood ratio tests based on a stepwise removal procedure (e.g., Jaeger, 2008). Additionally, participants and items were included as random effects to take into account their variability in each mixed-effects model. On Table 5 we report the coefficients in the final models thus identified, with *p*-values approximated by the normal distribution (Barr et al., 2013). Additionally, we run a series of mixed effects models on the L1 vs. L2 datasets separately, in order to test whether reading fluency, vocabulary and passage reading comprehension predicted accuracy to the sentence comprehension test to a different extent in the L1 vs. L2 groups.

**TABLE 5:** Summary of mixed effects models based on the accuracy to the sentence comprehension test.

	**β**	**SE**	***Wald Z***	***p***
**Group (L1 vs. L2)**				
Intercept	2.91	0.39	7.39	0.001
Group (L1 vs. L2)	–0.69	0.42	–1.65	0.09
**LSBQ Group (L1 vs. L2-dominant vs. L2-non-dominant)**				
Intercept	2.71	0.45	6.004	0.001
Group (L1 vs. L2-dominant)	0.15	0.48	0.32	0.75
Group (L1 vs. L2-non-dominant)	–1.25	0.51	–2.44	0.01^∗∗^
Group (L2-dominant vs. L2-non-dominant)	–1.07	0.54	–1.95	0.05^∗^

*Contrast were coded using dummy coding with L1 and L2-dominant used as reference levels. ^∗∗^Significant at the 0.01 level (two-tailed). ^∗^Significant at the 0.05 level (two-tailed).*

First, L1 students were more accurate than L2 in the sentence comprehension test, but this difference only approached significance (*p* = 0.09). However, interestingly, when we split the L2 group according to their relative dominance in L2, the picture changed drastically. As reported on the second section of Table 4, the group less dominant in L2 (L2-non-dominant) were 12% less accurate than L1 students and, on average, they produced 9% less correct responses than their peers that were L2-dominant. When we added group [χ^2^(3) = 6.45, *p* < 0.039] to the model, we observed that the L2-non-dominant group significantly differed both from L1 and L2-dominant. Importantly, the L2-dominant group showed a performance at the comprehension test comparable to that of L1 students.

We conducted a series of linear mixed effects models, based on the L1 and L2 data, respectively, to test whether accuracy to the sentence comprehension test was predicted by reading skills (word and non-word reading fluency, vocabulary skills, text comprehension, SES, chronological age, and, only for the bilingual dataset, length of exposure to the Italian language). For simplicity sake, we will report only significant results, omitting the χ^2^ values and the corresponding *p*-values when the predictors did not contribute to the fit of the model (and thus had to be excluded).

Regarding L1 data, accuracy to the sentence comprehension test was significantly predicted by word reading accuracy (Estimate = 0.084, SE = 0.370, *t* = 2.23, *p* < 0.02) and by vocabulary (Estimate = 0.030, SE = 0.014, *t* = 2.09, *p* < 0.03). When we considered the L2 data, the only significant predictors were non-word reading accuracy (Estimate = 0.211, SE = 0.96, *t* = 2.18, *p* < 0.03) and accuracy to the passage comprehension test (Estimate = 0.59, SE = 0.31, *t* = 1.89, *p* < 0.05). While it is perfectly reasonable that sentence comprehension (considered the fact that it involved reading) was significantly predicted by word reading and vocabulary, as appeared in the L1 group, it is remarkable that in the L2 dataset, sentence comprehension was predicted by non-word reading as well as by text comprehension. One might not exclude that, in this population, a task that requires reading underlies a sub-lexical decoding process, that is also involved in non-word reading. We will go back to these findings in the General Discussion.

### Comparison of L2-Non-dominant vs. L2-Dominant vs. L1 on Standardized Tests

**FIGURE 1:**
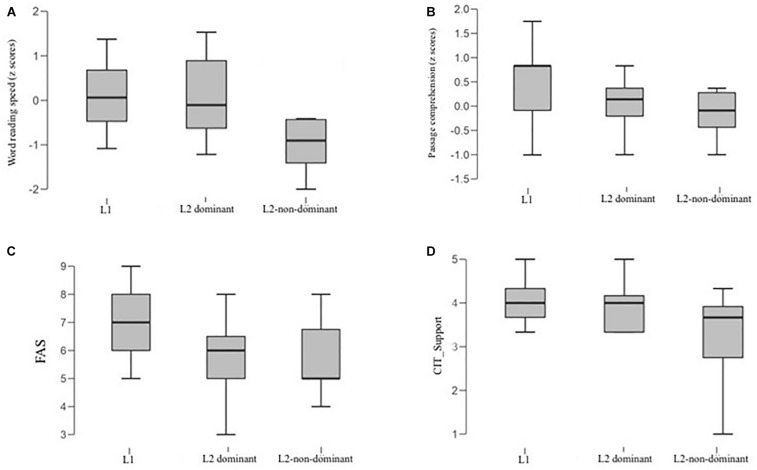
The four box plots represent the distribution of **(A)** word reading speed; **(B)** Passage reading comprehension; **(C)** scores to the FAS questionnaire on socioeconomic level; **(D)** CIT, Support scale as a function of group defined with respect to language dominance (i.e., L2 dominant vs. L2-non-dominant vs. L1).

Given the differences observed at the sentence comprehension test, we compared the performance of L2-non-dominant vs. L2-dominant vs. L1 on standardized tests by means of a series of Analysis of Variance using the Bonferroni correction. Inferential Statistics for each variable of the standardized tests are reported on Table 6 (F, *p*-values and *post hoc* tests) and graphically presented on Figure 1.

**TABLE 6:** Summary of the statistical analyses (Anova and *Post hoc* tests) of the assessment results by experimental group L1, L2-dominant and L2-non-dominant.

**Test**	***F*(1, 42)**	***p***	**η^2^**
Peabody	1.085	0.347	0.050

	***Post hoc* Test – *t***	***p* Bonf**	

L1 vs. L2-non-dominant	1.28	0.62	
L1 vs. L2-dominant	0.20	1.00	
L2-dominant vs. L2-non-dominant	–1.11	0.81	

	***F(*1, 42)**	***p***	**η^2^**

Passage reading comprehension (*z* scores)	3.255	0.049^∗^	0.137

	***Post hoc* Test – *t***	***p* Bonf**	

L1 vs. L2-non-dominant	2.400	0.063	
L1 vs. L2-dominant	–1.614	0.343	
L2- dominant vs. L2-non-dominant	0.785	1.000	

	***F(*1, 42)**	***p***	**η^2^**

Word reading accuracy (*z* scores)	8.540	< 0.001^∗∗^	0.294

	***Post hoc* Test – *t***	***p* Bonf**	

L1 vs. L2-non-dominant	3.917	0.001^∗∗^	
L1 vs. L2-dominant	–0.057	1.000	
L2- dominant vs. L2-non-dominant	3.441	0.004^∗∗^	

	***F(*1, 42)**	***p***	**η^2^**

Word reading speed (*z* scores)	8.044	0.001^∗∗^	0.282

	***Post hoc* Test – *t***	***p* Bonf**	

L1 vs. L2-non-dominant	3.885	0.001^∗∗^	
L1 vs. L2-dominant	–0.351	1.000	
L2- dominant vs. L2-non-dominant	3.167	0.009^∗∗^	

	***F(*1, 42)**	***p***	**η^2^**

Non-word reading accuracy (*z* scores)	0.495	0.486	0.012

	***Post hoc* Test – *t***	***p* Bonf**	

L1 vs. L2-non-dominant	3.885	0.001^∗∗^	
L1 vs. L2-dominant	0.005	1.000	
L2- dominant vs. L2-non-dominant	1.102	0.830	

	***F(*1, 42)**	***p***	**η^2^**

Non-word reading speed (*z* scores)	4.969	0.012^∗^	0.195

	***Post hoc* Test – *t***	***p* Bonf**	

L1 vs. L2-non-dominant	2.932	0.016^∗^	
L1 vs. L2-dominant	0.119	1.000	
L2- dominant vs. L2-non-dominant	2.711	0.029^∗^	

	***F(*1, 42)**	***p***	**η^2^**

FAS	5.469	0.008^∗∗^	0.215

	***Post hoc* Test – *t***	***p* Bonf**	

L1 vs. L2-non-dominant	2.632	0.036^∗^	
L1 vs. L2-dominant	–2.745	0.027^∗^	
L2- dominant vs. L2-non-dominant	–0.022	1.000	

	***F*(1, 42)**	***p***	**η^2^**

CIT (Support scale)	3.600	0.036^∗^	0.153

	***Post hoc* Test – *t***	***p* Bonf**	

L1 vs. L2-non-dominant	2.680	0.032	
L1 vs. L2-dominant	0.735	1.000	
L2- dominant vs. L2-non-dominant	1.718	0.281	

*^∗∗^Significant at the 0.01 level (two-tailed). ^∗^Significant at the 0.05 level (two-tailed).*

Regarding reading tests, there was a significant difference in word reading accuracy and speed, with L2-non-dominant being significantly slower than L2-dominant and L1, while no difference was found between L2-dominant and L1 groups. With respect to non-word reading, L2-dominant outperformed both L1 and L2-non-dominant, being significantly faster than both groups. L1 did not differ from L2-non-dominant. No differences were found with respect to non-word reading accuracy. Regarding passage reading comprehension, L2-non-dominant were significantly less accurate, as compared to L1; L2-dominant lay between the other groups and did not differ from any of them.

Importantly, L2-non-dominant were significantly below their peers also when considering the Support scale of the CIT, while no difference was found between L2-dominant and L1. Again, this represents an evidence of the fact that, within the L2 population, there might be students showing distress in the school context, and that only by inspecting their language profile it is possible to identify vulnerable students.

Interestingly, the only test where L2-dominant patterned similarly to L2-non-dominant was the FAS. That is, the two L2 groups showed a comparable SES, that resulted significantly below their L1 peers. This finding is extremely important because it suggests that, although coming from a context of relative socioeconomic disadvantage, this subgroup of L2 students showed a linguistic and learning performance, as well as a level of psychological well-being, basically overlapping with that of their L1 peers. It further indicates that the critical variable that allows to discriminate immigrant teens that might show a vulnerability in the school context is their inherent proficiency in L2.

## Discussion

Up to date, the lack of empirical studies on the language and learning profile of Italian L2 teenagers has made it difficult to clarify whether this population might be effectively considered at risk for school exclusion, as national reports seem to indicate (ISTAT, 2016). Therefore, to shed light on the nature of the problems that might characterize L2 adolescents, their learning profile as well as their language ability, the present study compared a group of L2 participants with a group of L1 peers that attended the same vocational school. L2 and L1 groups were tested on a series of standardized cognitive, linguistic and reading tests, as well as on an online sentence comprehension test. The test was created to identify subjects struggling with their L2.

Moving from the assumption that being proficient in the language of instruction might represent a protective factor for L2 students, we tested whether language comprehension difficulties in L2 might allow identifying students at risk for school exclusion, in accordance with other variables (i.e., SES, learning skills, and psychological well-being) that are known to be associated with this construct (e.g., Lam et al., 2014). We will first discuss results considering the L1 and L2 groups. Then, we will focus on the specific contribution of L2 proficiency in discriminating those students who might be at risk for school exclusion.

When comparing the experimental groups L1 and L2, results of the sentence comprehension test revealed that L2 were less accurate than the other group, but the difference did not reach significance. However, the analysis provided interesting results about the linguistic underpinnings of sentence comprehension process in monolingual and bilingual students. While in the L1 group, accuracy to the sentence comprehension task was predicted by vocabulary and word reading fluency, in the L2 sample, text reading comprehension as well as non-word reading speed were the only significant predictors.

These findings suggest that L2 students, when reading a sentence, tend to activate sub-lexical decoding, as well as higher-level mechanisms underlying text comprehension processes to comprehend it. Such findings offer an important clue about sentence comprehension mechanisms at stake in bilingual development. Namely, one possibility is that bilingual readers are more likely to rely on decoding processes, instead of lexical ones, due to a reduced L2 vocabulary size (though not significant in our study). Therefore higher-level processes underlying text comprehension are exploited only later, to make inferences about the internal structure of complex sentences.

Regarding standardized tests, the two groups behaved similarly only on non-word reading fluency and vocabulary skills. Conversely, there were significant differences in word reading fluency and passage reading comprehension scores. The fact that L2 students’ accuracy on word reading was significantly lower as compared to their monolingual peers, while non-word reading did not differ, confirmed that poorer reading performance in L2 students was not caused by a learning disability. Note additionally that in both groups, scores on reading tests, though significantly different, remained within the range of normality. Instead, this pattern of results indicated that bilinguals over-relied on sub-lexical mechanisms even at 15 years of age. However, sub-lexical decoding processes, while facilitating non-word reading, hampered word reading fluency.

This finding is in line with previous research based on early L2 Italian primary school children that outperformed their monolingual peers in non-word reading. In contrast, their ability in other reading and linguistic tasks appeared significantly lower with respect to monolinguals (Vernice and Pagliarini, 2018; see also Vender et al., 2016 for a comparable effect in pre-schoolers on non-word repetition). Note that it was not the aim of the current paper to disentangle whether word reading in L2 teens was based on lexical or sub-lexical mechanisms. Our results simply indicate that up to 15 years of age, bilingual students still rely on sub-lexical decoding in reading.

As for the family well-being, even though we tried to keep the SES as comparable as possible within the two groups, the L2 lived in a condition of socioeconomic disadvantage as compared to their monolingual peers. Additionally, when tested on a measure of psychological well-being in the classroom, L2 students were less likely to feel supported from the external environment.

We now discuss the contribution of L2 proficiency and use in order to better characterize the profile of students who could be at potential risk for school exclusion. Using a questionnaire aimed at testing language use in daily life contexts, we were able to identify two subgroups of L2 participants. The first one showed a higher use and exposure to Italian as compared to their L1 (L2-dominant group); the second one, in contrast, was characterized by use and exposure to both languages in different contexts (L2-non-dominant group). The L2-dominant group appeared to perform comparably to the L1 group in all standardized tests as well as on the sentence comprehension test. Conversely, the L2-non-dominant students proved to be significantly below their monolingual and bilingual (L2-dominant) peers in most of the standardized tests as well as on the sentence comprehension test.

As regards to psychological well-being, the CIT- Scale Support indicated that the L2-non-dominant group was significantly less likely to feel supported by the external context in comparison with L1, while L2-dominant did not differ from the other two groups. The L2-dominant group showed comparable scores with respect to L2-non-dominant group only regarding the SES. To sum up, the L2-non-dominant group appeared to be not only more impaired on most of learning and linguistic tasks in comparison with L1 and L2-dominant groups, but was also more vulnerable in terms of psychological well-being. Crucially, the co-occurrence of language and learning difficulties, together with the perception of scarce support within the external context, suggests that L2-non-dominant students are potential candidates for being at risk for school exclusion.

The striking difference we found on L2 participants based on proficiency, dominance and use of the L2, provides significant clues about the role of language dominance in determining the (linguistic and learning) profile of the bilingual speaker. Namely, we observed that language dominance exerts a critical effect in defining the linguistic and academic skills of the L2 student, and this finding is in line with previous studies indicating that dominance in a language, but not necessarily proficiency, determine the linguistic outcome in bilingual development (Perpiñán, 2017). Additionally, such finding reveals that, in line with our initial hypothesis, proficiency and use of the language of instruction represent a crucial asset in promoting effective inclusion in the classroom, exerting important implications for educational programs in high schools. Our data suggest that teachers and educators should be challenged to promote activities that enhance L2 proficiency targeting not only late bilinguals or newly arrived immigrants, but also L2 students who were born in Italy, showing a limited use of the majority language.

We are conscious of the fact that our L2-dominant speakers might be regarded as Heritage Language Speakers (henceforth, HLS). HLS are L2 speakers who have acquired their L1 simultaneously with the majority language (L2) or exclusively the L1 if the immigration has occurred during infancy (Benmamoun et al., 2013). They might use a minority language (their L1) in the home environment, though showing a predominant use and exposure in the daily life context to their L2 (Valdés, 2005). Research on bilingual speakers of minority languages has further shown that HLS might fail to acquire full linguistic competence in the heritage language, i.e., their L1 (Polinsky and Kagan, 2007). As a consequence, in adulthood, linguistic outcomes highlight atypical acquisition patterns (Kupisch and Rothman, 2016). Importantly, in our study, we observed that L2-dominant participants showed a distinctive profile as compared to their bilingual and monolingual peers. These students tended to use their L2, Italian, to a greater extent in daily life, as compared to their L2-non-dominant peers. However, given the fact that, in order to properly define HLS, one needs to collect information about the real language proficiency in L1, we might not be sure that L2-dominant can be regarded as HLS. For this reason, we safely categorized the groups based on use and exposure to L2 language.

With respect to the current study, it is important to discuss a number of potential limitations. First, finding a homogenous sample of participants in terms of first age and length of exposure to L2, implied trimming a consistent number of L2 students that attended the vocational high school involved in the study. As a consequence, the current research, given the small sample of participants, offers only an initial contribution to the study of the relation between weakness in the L2 and risk for school exclusion. We are conscious that our results do not allow to draw conclusions on the population of teens of immigrant families in Italy. Thus, in future work, we aim to extend our sample size, ideally involving a consistent number of participants with diverse L1 backgrounds. By doing so, it would be possible to investigate, for instance, gender and cultural differences between subgroups.

A second aspect that we were not able to take into account in the current research, but that is deeply intertwined with the first point discussed above, refers to the inclusion of a measure of academic achievement. We believe that this variable could provide additional information about cross-cultural differences in academic attainment and motivation to learn (Bempechat and Drago-Severson, 1999). Additionally, an objective measure of school achievement would clarify whether the sentence comprehension test positively correlates with school outcomes, at least in disciplines where reading comprehension is crucial. Given the importance of this point, it would be worth further investigation.

Third, regarding the sentence comprehension test, we deliberately opted for a simple scoring procedure. We are aware of the fact that there exist alternative ways to score the current data. For instance, one could assign intermediate scores to compatible sentences or penalize a contradictory response with a negative score. Alternatively, it would be even possible to transform raw scores according to the Item Response Theory approach (IRT; Hambleton and Jones, 1993). By doing so, the test would allow to assess the ability of each student in the sentence comprehension test with respect to the difficulty of the individual test items. However, since the current study represents only an initial phase of our research, we preferred to follow the simplest scoring procedure. Recall that the current test is not meant to offer a diagnostically sophisticated measure of linguistic competence, rather to provide a quick screening task to be used by teachers and educators in schools. It is possible that, in future steps of our research, we might develop a more complex scoring procedure for the sentence comprehension test.

## Conclusion

In conclusion, we observed that, in a sample of early bilinguals, the relative disadvantage of some L2 students at the linguistic and academic level was large. The linguistic problems were importantly captured by a rapid but efficient test of sentence comprehension, that we created on purpose. The sentence comprehension test exerted several advantages: first, it could be carried out by teachers or educators following simple instructions, in contrast to tests of reading fluency and comprehension that must be administered by health professionals. Second, the possibility to use it in schools as an on-line tool would allow to address the urgent need of teachers to adequately but quickly identifying those students that are struggling with sentence comprehension.

To sum up, our study indicates that an intervention aimed at targeting the sentence comprehension ability may help teachers and educators to assess the language skills of students. In fact, language proficiency is of greatest importance for personal and academic realization, and, as we observed in the current study, it represents a critical variable in signaling students who might manifest a vulnerability within the school context. By using a simple online tool, such as the sentence comprehension test presented here, readily available to educators, teachers might offer adequate educational opportunities to all the students, promoting cohesion in the classroom.

## Data Availability Statement

The datasets generated for this study are available on request to the corresponding author.

## Ethics Statement

The studies involving human participants were reviewed and approved by the Ethics Committee of Catholic University of the Sacred Heart. Written informed consent to participate in this study was provided by the participants’ legal guardian/next of kin.

## Author Contributions

MV: creation of the “Sentence Comprehension Test” with MM conception and design of the work, data collection, data analysis, interpretation, and drafting the manuscript. MM: conception and creation of the “Sentence Comprehension Test”. MT: data collection and coding, and drafting of the section Materials and Methods. MC and EL: data collection and coding. DS: conception of the design and critical revision of the draft. MG and ML: conception of the “Sentence Comprehension Test”. All the authors contributed to the critical revision of the manuscript.

## Conflict of Interest

The authors declare that the research was conducted in the absence of any commercial or financial relationships that could be construed as a potential conflict of interest.
