# Supplementary material for: An Online Tool to Assess Sentence Comprehension in Teenagers at Risk for School Exclusion: Evidence From L2 Italian Students
Source: Front Psychol. 2019 Nov 5;10:2417. doi: 10.3389/fpsyg.2019.02417 (PMC6849484; doi:10.3389/fpsyg.2019.02417)
Supplement: Supplementary file 1 [file Data_Sheet_1.doc]

**Appendix A**

Below we report the 20 target sentences of the sentence comprehension test and the four response options (a: equivalent; b: contradictory; c: compatible; d: distractor). When the test is administered, the four options are randomized. English translation in brackets.

Q1. Gianni è in grado di correre per un'ora intera come lo era dieci anni fa.

[Gianni is able to run for a whole hour as he was ten years ago.]

1. Dieci anni fa Gianni poteva correre per un'ora intera e riesce a farlo anche adesso.

[Ten years ago Gianni could run for a whole hour and he can do it even now.]

1. Dieci anni fa Gianni correva per un'ora intera ma adesso non è più in grado di farlo.

[Ten years ago Gianni used to run for a whole hour but now he is no longer able to do it.]

1. Gianni riesce a camminare per un'ora come dieci anni fa.

[Gianni can walk for an hour like ten years ago.]

1. Correre per un'ora fa ringiovanire di dieci anni.

[Running for an hour makes one ten years younger.]

Q2. Per Mario è più importante realizzare ciò che desidera che essere leale.

[For Mario it is more important to desire what he wants then to be fair.]

1. Per Mario conta maggiormente realizzare i suoi sogni che comportarsi onestamente.

[For Mario it is more important to realize his dreams than to behave honestly.]

1. Mario antepone la lealtà ai propri desideri.

[Mario puts loyalty before his wishes.]

1. Mario realizza spesso i propri desideri.

[Mario often fulfills his desires.]

1. Le persone leali si impegnano a fondo per realizzare ciò che desiderano.

[Loyal people work hard to achieve what they want.]

Q3. Giulia si sente in imbarazzo ad offrirsi volontaria ad una interrogazione, anche se gli insegnanti lo apprezzano.

[Giulia feels embarrassed to volunteer for an oral test, even if the teachers

appreciate it.]

1. I professori giudicano positivamente che Giulia si offra per un'interrogazione, ma lei ne è imbarazzata.

[Teachers evaluate positively that Giulia volunteer for an oral test, but she is embarrassed by it.]

1. Gli insegnanti non apprezzano gli studenti che si offrono volontari alle interrogazioni e questo mette in difficoltà Giulia.

[Teachers do not appreciate students who volunteer for an oral test and this puts Giulia in trouble.]

1. Giulia si sente in difficoltà a svolgere le verifiche scritte, anche quando ha studiato.

[Giulia encounters difficulties to carry out written tests, even when she has studied.]

1. I compagni di classe apprezzano coloro che si offrono volontari ad un'interrogazione.

[Classmates appreciate those who volunteer for an oral test.]

Q4. In nessuna delle aule agli studenti è permesso consumare il proprio pasto.

[In none of the classrooms, students are allowed to eat their own meal.]

1. In tutte le aule agli studenti è vietato mangiare il loro pasto.

[In every classroom, students are forbidden to eat their meal.]

1. Agli studenti è consentito mangiare dovunque essi vogliano.

[Students are allowed to eat wherever they want.]

1. All'interno delle aule gli studenti possono sedersi dove desiderano.

[In the classroom students can sit where they wish.]

1. Gli studenti possono mangiare in mensa oppure nei bar vicino all'università.

[Students can eat at the cafeteria or at the coffee shops near the university.]

Q5. A volte Luca non riesce a pensare a nient'altro che a quanto gli piacerebbe mangiare

un piatto di cannelloni.

[Sometimes Luca cannot think of anything else than eating cannelloni.]

1. A volte Luca pensa solo a quanto gli piacerebbe mangiare i cannelloni.

[Sometimes Luca thinks exclusively about how much he would like to eat cannelloni.]

1. Luca non pensa mai a quanto gli piacerebbe mangiare i cannelloni.

[Luca never thinks about how much he would like to eat cannelloni.]

1. A volte Luca si distrae pensando a quanto gli piacerebbe mangiare una torta.

[Sometimes Luca is distracted thinking about how much he would like to eat a cake.]

1. Luca a volte si cucina un piatto di cannelloni.

[Luca sometimes cooks a plate of cannelloni for himself.]

Q6. Spesso il professore ha sentito gli studenti protestare quando ha cercato di evitare

che copiassero i compiti.

[The professor often heard students complain when he tried to prevent them

from copying tests.]

1. Frequentemente, quando il professore cerca di impedire agli studenti di copiare i compiti, questi protestano.

[Students frequently protest when the professor tries to prevent them from copying tests.]

1. Spesso, quando il professore prova ad impedire agli alunni di copiare i compiti, gli studenti ubbidiscono.

[When the professor tries to prevent the students from copying tests, students often obey.]

1. Spesso, quando il professore prova ad impedire agli studenti di copiare i compiti, questi parlano.

[When the professor tries to prevent the students from copying tests, they often talk.]

1. Spesso, quando il professore prova ad impedire agli studenti di fumare in classe, li sente protestare.

[When the professor tries to prevent students from smoking in the classroom, he often hears them protest.]

Q7. Sara ha criticato alcuni bambini che cercavano di imbrattare i muri, non perché

gliene importasse molto, ma per una questione di principio.

[Sara criticized some children trying to smear the walls, not because she cared about

it so much, but as a matter of principle.]

1. Sara ha sgridato dei bambini che tentavano di sporcare i muri perché non si deve fare e non perché questo la riguardasse direttamente.

[Sara scolded children who tried to dirty the walls because this should not be done and not because it was directly related to her.]

1. Nessun bambino che cercava di imbrattare i muri è stato rimproverato da Sara per una questione di principio.

[No child trying to smear the walls was scolded by Sara as a matter of principle.]

1. Sara ha sgridato alcuni bambini che cercavano di scavalcare un muro perché aveva paura che si facessero male.

[Sara scolded some children who tried to climb over a wall because she was afraid they would get hurt.]

1. Sara ha sgridato alcuni bambini che cercavano di imbrattare i muri perché aveva paura che arrivasse la polizia.

[Sara scolded some children who tried to smear the walls because she was afraid the police would come.]

Q8. Se Mauro trova vecchi amici o persone che non incontra da molto tempo, fa finta di

niente, a meno che non siano questi i primi a salutarlo.

[If Mauro runs into old friends or someone he hasn’t met for a very long time, he

pretends nothing happened unless they wave at him first.]

1. Mauro fa finta di niente quando incontra vecchi amici o persone che non vede da molto tempo, a meno che non siano loro i primi a salutarlo.

[Mauro pretends nothing happened when he meets old friends or people he has not seen for a long time, unless they wave at him first.]

1. Quando Mauro incontra vecchi amici o persone che non vede da molto tempo li saluta sempre per primo.

[When Mauro meets old friends or people he has not seen for a long time he always waves at them first.]

1. Mauro saluta calorosamente gli amici solo se sono questi i primi a salutarlo.

[Mauro warmly greets his friends only if they greet him first.]

1. Gli amici di Mauro lo salutano sempre per primi, anche se lui fa finta di niente.

[Mauro's friends always greet him first, even if he pretends nothing happened.]

Q9. A tavola se c'è qualcosa che Rosa deve mangiare ma che in realtà non vuole

mangiare, mangia molto lentamente nella speranza di non doverla finire.

[If there is something an the table that Rosa has to eat but that she does not want to eat, she eats really slowly hoping to avoid eating it all.]

1. Quando a tavola Rosa deve mangiare qualcosa che non vuole, mangia molto adagio, sperando di non doverla finire.

[At the table, when Rosa has to eat something she does not want to, she eats very slowly, hoping to avoid eating it.]

1. Rosa mangia sempre velocemente ciò che non vuole ma deve mangiare.

[Rosa always eats fast what she does not want but has to eat.]

1. Rosa mangia ciò che deve e che vuole mangiare.

[Rosa eats what she has to and wants to eat.]

1. Rosa deve bere molto, anche se in realtà non le piace.

[Rosa has to drink a lot, even if she does not really like it.]

Q10. Giovanni non è mai contento a meno che non sia in vacanza o allo stadio.

[Giovanni is never happy unless he is on vacation or at the stadium.]

1. Giovanni è contento soltanto quando si trova in vacanza o allo stadio.

[Giovanni is happy only when he is on vacation or at the stadium.]

1. Giovanni non è mai felice quando è in vacanza o allo stadio.

[Giovanni is never happy when he is on vacation or at the stadium.]

1. Giovanni si diverte a guardare una partita di calcio allo stadio.

[Giovanni enjoys watching a soccer game at the stadium.]

1. Giovanni va allo stadio nei giorni di vacanza.

[Giovanni goes to the stadium on vacation.]

Q11. Gianni non è in grado di obbligare sé stesso a smettere di mangiare caramelle.

[Gianni is unable to force himself to stop eating sweets.]

1. Sforzarsi di non mangiare le caramelle è impossibile per Gianni.

[It is impossible for Gianni making an effort not to eat candies.]

1. Gianni può imporsi di smettere di mangiare le caramelle.

[Gianni can impose himself to stop eating candies.]

1. Gianni è in grado di controllarsi nel bere.

[Gianni is able to control his drinking.]

1. Bisognerebbe che ognuno obbligasse sé stesso a smettere di mangiare caramelle.

[Everyone should force himself to stop eating candies.]

Q12. Benché Lucia non sia soddisfatta del vestito che ha comprato, non c'era nient'altro

che le stesse bene.

[Although Lucia is not satisfied with the dress she bought, there was nothing else

that looked good on her.]

1. Lucia ha comprato l'unico vestito che le stava bene anche se non ne era completamente soddisfatta.

[Lucia bought the only dress that looked good on her even though she was not completely satisfied.]

1. Lucia ritiene che il vestito che ha comprato le stia davvero bene.

[Lucia believes that the dress she bought looks really good on her.]

1. Lucia ha comprato un vestito che le stava bene e lo metterà alla festa.

[Lucia bought a dress that looked good on her and will wear it at the party.]

1. Lucia non ha comprato delle scarpe che le stavano bene.

[Lucia did not buy shoes that looked good on her.]

Q13. Luca non si diverte a chiacchierare con le persone quando non è a suo agio con loro.

[Luca does not enjoy chatting with people when he is not comfortable with them.]

1. Se Luca non si sente a suo agio con qualcuno, non ci parla con piacere.

[If Luca does not feel comfortable with someone, he does not talk to them with pleasure.]

1. Quando Luca è a disagio con qualcuno, si diverte a parlare con lui.

[When Luca is uncomfortable with someone, he enjoys talking to them.]

1. Se Luca si trova bene con una persona, la ascolta volentieri.

[If Luca feels comfortable with a person, he is happy to listen to him.]

1. Se uno non si diverte a chiacchierare con le persone vuol dire che non le sa ascoltare.

[If someone does not enjoy chatting with people, it means that he cannot listen to them.]

Q14. Una volta che Piero ha comprato un regalo per qualcuno, trova difficile credere che

possa aver fatto una scelta sbagliata.

[Once Piero bought a gift for someone, he finds hard to believe he may have made the

wrong choice.]

1. Dopo che Piero ha comprato un regalo per qualcuno, non riesce a pensare che la sua scelta è stata sbagliata.

[After Piero bought a gift for someone, he cannot think that his choice has been wrong.]

1. Una volta che Piero ha comprato qualcosa per qualcuno, è probabile che pensi di aver fatto una scelta non adatta.

[Once Piero has bought something for someone, it is likely he thinks he made an inappropriate choice.]

1. Quando Piero compra qualcosa per sua moglie, non riesce ad ammettere di aver fatto una scelta sbagliata.

[When Piero buys something for his wife, he cannot admit he made the wrong choice.]

1. Prima di comprare un regalo, Piero trova difficile credere di aver fatto una scelta che sarà apprezzata.

[Before buying a gift, Piero finds hard to believe that he made a choice that will be appreciated.]

Q15. La maggior parte delle persone commetterebbe delle scorrettezze per ottenere un

beneficio o per evitare di perderlo.

[Most people would behave inappropriately to take advantage of something or to avoid losing it.]

1. Molta gente è disposta a fare cose disoneste per trarne un vantaggio o mantenerlo.

[Many people are willing to do dishonest things to have an advantage or to keep.]

1. Per ottenere un beneficio o mantenerlo molta gente si comporterebbe perfino in modo leale.

[To get a benefit or keep it, lots of people would even behave fairly.]

1. Molte persone potrebbero venir meno ai loro principi per preservare o ottenere benefici per i propri familiari.

[Many people may go against their principles to preserve or obtain benefits for their family members.]

1. La maggior parte dei benefici deriva dall'aver commesso una scorrettezza.

[Most benefits come from having behaved inappropriately.]

Q16. Lina è triste quando le scarpe che indossa più volentieri devono essere mandate dal calzolaio a riparare.

[Lina is sad when the shoes she wears more willingly must be sent by the shoemaker

to repair.]

1. Lina è triste quando le sue scarpe preferite devono essere mandate in riparazione dal calzolaio.

[Lina is sad when her favorite shoes have to be sent to the shoemaker to be repaired.]

1. Lina è felice quando le sue scarpe preferite vengono mandate dal calzolaio per essere riparate.

[Lina is happy when her favorite shoes are sent to the shoemaker to be repaired.]

1. Lina è triste quando le sue scarpe preferite devono essere lucidate.

[Lina is sad when her favorite shoes need to be polished.]

1. Lina indossa un paio di scarpe che devono essere riparate dal calzolaio.

[Lina wears a pair of shoes which must be repaired by the shoemaker.]

Q17. Luca sta attento a tutti gli indizi che dimostreranno che i suoi sospetti sono

giustificati.

[Luca pays attention to all the evidence showing his suspicions are justified.]

1. Per giustificare i suoi sospetti, Luca esamina con cura tutti gli indizi.

[To justify his suspicions, Luca examines all the clues carefully.]

1. Per non dare peso ai suoi sospetti, Luca sorvola su qualunque possibile indizio.

[In order not to give weight to his suspicions, Luca flies over any possible clue.]

1. Luca considera dettagli significativi per convincersi dei suoi sospetti.

[Luca evaluates substantial details to convince himself of his suspicions.]

1. Solo indizi certi dimostreranno che i sospetti di Luca sono giustificati.

[Only certain clues will show Luca's suspicions are justified.]

Q18. Se Marco trovasse lo smartphone di qualcuno, non si sentirebbe a disagio a leggerne

i messaggi.

[If Marco found someone's smartphone, he wouldn't feel uncomfortable reading their

messages.]

1. Se Marco trovasse lo smartphone di qualcuno, non si sentirebbe in colpa a leggerne i messaggi.

[If Marco found someone's smartphone, he wouldn't feel guilty reading the messages.]

1. Se Marco trovasse lo smartphone di qualcuno, sarebbe in imbarazzo a leggerne i messaggi.

[If Marco found someone's smartphone, he would be embarrassed to read the messages.]

1. Se Marco trovasse lo smartphone di qualcuno, cercherebbe subito il proprietario per restituirglielo.

[If Marco found someone's smartphone, he would immediately look for the owner to return it to him.]

1. Marco si è sentito a disagio a leggere i messaggi nello smartphone che ha trovato.

[Marco felt uncomfortable reading the messages on the smartphone he found.]

Q19. Elena non sarebbe tranquilla se qualcuno dei suoi amici si trovasse in difficoltà con

un esame.

[Elena could not relax if any of her friends were in trouble with an exam.]

1. Se un amico di Elena facesse fatica con un esame, lei sarebbe in pensiero per lui.

[If a friend of Elena were struggling with an exam, she would be worried about him.]

1. Se tra gli amici di Elena ce ne fosse qualcuno in difficoltà con un esame, a lei non importerebbe molto.

[If any of Elena's friends were in trouble with an exam, she would not care much.]

1. A Elena importa sapere che i suoi amici stanno bene.

[Elena cares about her friends being fine.]

1. Se uno si trovasse in difficoltà con un esame non potrebbe essere tranquillo.

[If one were struggling with an exam, he could not relax.]

Q20. Gino non cerca di nascondere la stima che sente per i suoi amici, per fare in modo che si rendano conto dei suoi sentimenti.

[Gino does not try to hide the respect he feels for his friends to make them realize his feelings.]

1. Per fa sì che i suoi amici si rendano conto di quello che prova per loro, Gino manifesta apertamente l'apprezzamento che nutre nei loro confronti.

[To ensure that his friends realize what he feels for them, Gino openly expresses his appreciation he harbors for them.]

1. Gino non vuole offendere i suoi amici, e per questo cerca sempre di nascondere quello che prova per loro.

[Gino does not want to hurt his friends and, because of that, he always tries to hide what he feels for them.]

1. Per essere sincero con i suoi amici, Gino fa vedere che li apprezza.

[To be honest with his friends, Gino shows them his approval.]

1. Gino manifesta quel che prova per i suoi amici, per essere orgoglioso dei suoi sentimenti.

[Gino shows what he feels for his friends in order to be proud of his feelings.]
